# Can ginger ameliorate chemotherapy-induced nausea? Protocol of a randomized double blind, placebo-controlled trial

**DOI:** 10.1186/1472-6882-14-134

**Published:** 2014-04-09

**Authors:** Wolfgang Marx, Alexandra L McCarthy, Karin Ried, Luis Vitetta, Daniel McKavanagh, Damien Thomson, Avni Sali, Liz Isenring

**Affiliations:** 1 Centre of Dietetics Research, University of Queensland, St Lucia, Brisbane, QLD, Australia; 2 National Institute of Integrative Medicine, Melbourne, VIC, Australia; 3 Division of Cancer Services, Princess Alexandra Hospital, Brisbane, QLD, Australia; 4 Institute of Biomedical Innovation, Queensland University of Technology, Brisbane, QLD, Australia; 5 School of Medicine, Princess Alexandra Hospital, Brisbane, QLD, Australia; 6 Medlab, Sydney, NSW, Australia; 7 Oncology & Haematology Unit, Princess Alexandra Hospital, Brisbane, QLD, Australia; 8 PAH Oncology & Haematology Unit, Princess Alexandra Hospital, Brisbane, QLD, Australia; 9 Department of Nutrition & Dietetics, Princess Alexandra Hospital, Brisbane, QLD, Australia; 10 Health Sciences & Medicine, Bond University, Brisbane, QLD, Australia

**Keywords:** Ginger, CINV, Nausea

## Abstract

**Background:**

Preliminary research shows ginger may be an effective adjuvant treatment for chemotherapy-induced nausea and vomiting but significant limitations need to be addressed before recommendations for clinical practice can be made.

**Methods/Design:**

In a double–blinded randomised-controlled trial, chemotherapy-naïve patients will be randomly allocated to receive either 1.2 g of a standardised ginger extract or placebo per day. The study medication will be administrated as an adjuvant treatment to standard anti-emetic therapy and will be divided into four capsules per day, to be consumed approximately every 4 hours (300 mg per capsule administered q.i.d) for five days during the first three cycles of chemotherapy. Acute, delayed, and anticipatory symptoms of nausea and vomiting will be assessed over this time frame using a valid and reliable questionnaire, with nausea symptoms being the primary outcome. Quality of life, nutritional status, adverse effects, patient adherence, cancer-related fatigue, and CINV-specific prognostic factors will also be assessed.

**Discussion:**

Previous trials in this area have noted limitations. These include the inconsistent use of standardized ginger formulations and valid questionnaires, lack of control for anticipatory nausea and prognostic factors that may influence individual CINV response, and the use of suboptimal dosing regimens. This trial is the first to address these issues by incorporating multiple unique additions to the study design including controlling for CINV-specific prognostic factors by recruiting only chemotherapy-naïve patients, implementing a dosing schedule consistent with the pharmacokinetics of oral ginger supplements, and independently analysing ginger supplements before and after recruitment to ensure potency. Our trial will also be the first to assess the effect of ginger supplementation on cancer-related fatigue and nutritional status. Chemotherapy-induced nausea and vomiting are distressing symptoms experienced by oncology patients; this trial will address the significant limitations within the current literature and in doing so, will investigate the effect of ginger supplementation as an adjuvant treatment in modulating nausea and vomiting symptoms.

**Trial registration:**

ANZCTR.org.au Identifier: ACTRN12613000120774.

## Background

### Chemotherapy-induced nausea and vomiting places a significant burden on the patient

Despite the efficacy of cytotoxic interventions in the treatment of cancer, these treatments are often accompanied by a variety of adverse effects. Chemotherapy-induced nausea and vomiting (CINV) is a relatively common side effect of this treatment and has been repeatedly rated as one of the most distressing symptoms in this setting [1,2]. While there have been multiple classes of medications developed to treat this symptom, nausea and vomiting persists in a large number of patients. The incidence of vomiting has been significantly reduced through combinations of anti-emetic medications, but efforts to control nausea have been less successful. Affecting upwards of 60% of patients [3], CINV has also been shown to significantly impact on patient quality of life (QoL). Moreover, although it happens rarely, CINV can be so severe that it can lead to dose reduction or treatment discontinuation, and subsequently increase the risk of disease progression [3-5].

### Ginger extract appears beneficial in treating chemotherapy-induced nausea and vomiting

Ginger (*Zingiber officinale*) has been used for centuries by many cultures as a folk-remedy for gastrointestinal-related conditions [6]. Previous clinical trials have provided support for its use in the treatment of nausea in multiple settings including CINV [7-9] and two previous clinical trials have found ginger supplementation to be as effective as metoclopramide in reducing symptoms of CINV [10,11]. Furthermore, animal and cell culture data have demonstrated a viable mechanism of action for its anti-nausea effect [12].

The rhizome of ginger possesses an array of bioactive compounds (i.e. gingerols, shogaols, zingiberene, zingerone, and paradol) that may be responsible for the reported beneficial effects of ginger use. Cell culture and animal studies suggest that these constituents stimulate oral and gastric secretions [13], regulate gastrointestinal motility [14,15], interact with the 5-HT_3_ and NK-1 receptors implicated in the CINV reflex [16,17], and assist in rescuing intracellular redox metabolism [18]. Of note, the interaction of ginger with 5-HT_3_ and NK-1 receptors is particularly promising as the success of modern anti-emetic medications (i.e. 5-HT_3_ and NK-1 antagonists) are due to similar interactions with these same receptors. Furthermore, animal studies provide preliminary support for the role of ginger supplementation in the prevention of cisplatin-induced emesis [19,20].

A recent review found seven clinical studies have investigated ginger supplementation in this setting [21]. These studies present a contentious picture of the efficacy of ginger as an anti-CINV treatment in patients administered chemotherapy, with three demonstrating a positive effect, two in favour but with caveats, and two reporting no effect on measures of CINV. However, multiple limitations were identified within the existing literature that need to be resolved before clinical recommendations can be made.

### Chemotherapy-induced nausea and vomiting poses a significant risk to patients’ nutritional status and treatment outcomes

Previous studies report that approximately 50% of patients in the oncology setting are malnourished [22]. Malnutrition is a serious concern for oncology patients as it can significantly and severely affect QoL and treatment-related outcomes. Malnutrition can result in compromised immune function, reduced functional status, decreased performance status, and impaired treatment response [23-25]. Nausea and vomiting in this setting are of significant concern in patients diagnosed with cancer, as these symptoms can adversely affect dietary intake, increasing the risk of malnutrition during treatment.

It is feasible then to suggest that interventions that improve nausea and vomiting during chemotherapy may consequentially aid in improving and maintaining nutritional status. However, to date, there are no studies that have investigated the influence of ginger on patient nutritional status in this setting. Therefore, this protocol assesses nutritional status after each cycle using the validated questionnaire, the Patient Generated- Subjective Global Assessment, performed by an accredited dietitian.

### Chemotherapy-induced nausea and vomiting might exacerbate or be physiologically related to chemotherapy-related fatigue

Like nausea and vomiting, chemotherapy-related fatigue (CRF) is both highly prevalent in this population and can significantly influence the patient’s quality of life [26,27]. The results from a number of studies that have investigated CRF have found nausea and vomiting to be a strongly associated set of symptoms [28]. The reason for this is not fully elucidated but due to this significant correlation, treatment options that have been traditionally targeted at treating CINV should be further investigated as these modalities may also provide benefit to patients experiencing CRF. Using the Functional Assessment of Chronic Illness Therapies- Fatigue (FACIT-F) subscale, our study will be the first to investigate the effect of adjuvant ginger supplementation on self-reported measures of fatigue.

### Comprehensive, validated questionnaires are required to assess chemotherapy-induced nausea and vomiting

In order to assess CINV, the instrument used needs to be able to accurately capture the relevant aspects of CINV. Nausea, vomiting and retching, while temporally related, are distinct phenomena and therefore, are required to be measured as separate entities. In addition to this, a well-developed questionnaire should be able to provide a detailed picture of each phenomena. Widely used questionnaires in this setting include questions that measure multiple domains of CINV such as the severity, the perceived intensity of CINV; frequency, the amount of times CINV occurred over a time period; duration, the length of time that these symptoms persisted; and distress, the perceived burden that these symptoms place on the patients daily function and QoL [29].

There have been several questionnaires developed for the use of measuring nausea and vomiting, not only in the chemotherapy setting but also in other areas. A recent review identified 25 instruments that have been developed to measure nausea and vomiting in the clinical setting [29]. The authors used a list of criteria to determine the scope of nausea and vomiting that each questionnaire was able to capture. Of all questionnaires reviewed, no one tool fulfilled all criteria; however, the Index of Nausea, Vomiting, and Retching (INVR) tool was found to best meet this criteria [30].

A recent review found that only one previous study that investigated the effect of ginger on CINV used the INVR questionnaire [21]. This poses a significant limitation to the current literature as it is plausible that in these previous studies, ginger may have provided some benefit to domains of CINV that were not captured by the questionnaires employed in these respective studies.

Therefore, in order to ensure that our study is able to capture all relevant factors involved in CINV, it is important to use a questionnaire that is both validated and comprehensive and so it was decided that this study will incorporate the INVR questionnaire.

### Predisposing factors influence individual susceptibility to chemotherapy-induced nausea and vomiting

Multiple factors are reported to influence the individual risk of a patient developing CINV [31]. These factors relate not only to the treatment protocol but also the patient’s lifestyle, mental state, and previous experience with nausea and vomiting in other settings [32-34]. Consequently, while the emetogenicity of the treatment protocol is the major determinant of CINV risk, a patient with multiple predisposing factors can experience significant levels of CINV despite being prescribed a low emetogenic chemotherapy regimen. Of particular concern is the development of anticipatory nausea and vomiting, a conditioned response that is difficult to treat, and the gradual resistance to anti-emetic therapy after multiple chemotherapy cycles [35].

These factors represent a significant set of potential confounding variables for RCTs in this setting. To date, all trials in this area have recruited patients that have already experienced nausea and vomiting in previous chemotherapy cycles. This allows for the potential recruitment of patients with an already established resistance to additional anti-emetic therapies. Furthermore, if lifestyle factors such as alcohol intake and previous experience of motion sickness, which have been shown to influence CINV risk, are not screened for, this may result in a study comprised of two groups with a predisposed heterogeneous response to CINV. To date, this has not been thoroughly controlled for and therefore, may account for some of the difference in the results between previous trials. We have developed a short questionnaire that aims to assess these factors and will be the first study to factor this into our post-study statistical analysis.

### Previous dosing regimens and formulations of ginger may not have been optimal

In two recent studies that investigated the pharmacokinetics of multiple ginger compounds, it was found that these compounds have a relatively short half-life of approximately 1.5-3 hours [36,37]. In order to ensure that there are sufficient plasma levels of the active compounds throughout the day, the dosage in this study is divided between 4 capsules that will be consumed approximately every 4 hours.

The dosage of 1.2 g was selected for the following reasons: 1) it is within the typical dosage utilised in previous literature; 2) a lower dose, divided into multiple capsules, might not reach adequate concentrations to be effective; and 3) concerns that higher doses would reduce CINV control. Previous studies indicated higher doses were either less effective or possibly interfered with standard anti-emetic medications [38].

An additional limitation in the existing literature is the inconsistent use of standardized ginger extracts. Of the seven studies included in a recent review, only two studies used a ginger formulation that had been standardised to the relevant bioactive compounds while the remaining five used a crude ginger powder in capsule form [21]. The concentration of active compounds found within preparations of ginger has been found to be highly variable and can be influenced by the storage, location, and type of processing involved in the manufacturing of a specific ginger product [39]. Due to the majority of previous studies using unstandardized formulations, the inconsistent results reported in the literature may be attributed to the differences in compounds the formulations used in each study.

To control for this limitation, we are using a ginger extract that has been standardised to contain 5% gingerols. We have also arranged for a sample of our ginger capsules to be independently analysed at the commencement and completion of our study to ensure the potency of the formulation.

Incorporating the results of these studies, we are expanding on the current literature as the majority of previous trials have used dosing regimens that are inconsistent with these findings.

### Purpose of study and objectives

#### Purpose of study

Despite advances in anti-emetic medication, CINV continues to be a significant problem for many patients undergoing chemotherapy and is often rated as one of the most deleterious side-effects of cancer chemotherapeutic treatments. There is evidence from international trials that ginger formulations, in conjunction with standard anti-emetic medication, can be effective in the treatment of CINV. However, this therapy is not routinely used in oncology clinics due to its novelty and the lack of information about how patients will tolerate ginger in the clinical setting.

### Hypothesis

It is hypothesised that in chemotherapy-naïve medical oncology patients about to commence treatment of any emetogenicity, adjuvant ginger supplementation compared with placebo will:

1. Reduce the frequency, distress and duration of chemotherapy-induced nausea (i.e. acute, delayed and anticipatory) during each chemotherapy cycle (up to 3 cycles).

2. Reduce frequency, distress and duration of chemotherapy-induced vomiting and retching

3. Result in improved nutritional status, physical function and quality of life

4. Be adhered to (>80% consumption of supplements) and well tolerated (no significant adverse events related to ginger supplementation).

### Outcomes

#### Primary outcomes

• The frequency, severity, duration of acute and delayed nausea

### Secondary outcomes

• The frequency and severity of acute and delayed vomiting

• The frequency and severity of acute and delayed retching

• Change in ratings of cancer-related fatigue

• Adequacy of supplement blinding

• Change in nutrition status

• Incidence and severity of symptoms associated with treatment

• Change in quality of life

• Change in quality of life caused by nausea and vomiting

• Patient adherence to intervention

• Influence of previously identified factors that affect the generation of CINV

### Investigational plan

#### Overall study design

This study will be a double-blinded, randomised, placebo-controlled trial. Outcomes will be assessed at three days prior to chemotherapy, one day prior to chemotherapy, on the day of chemotherapy, and during the 4 days post-chemotherapy. Participants will consume the study medication for 5 days per chemotherapy cycle, commencing on the day of chemotherapy. This will be repeated over 3 chemotherapy cycles.

### Setting

The trial will be initially conducted at the Princess Alexandra Hospital, Brisbane, Australia. Additional sites will be utilised if further funding is obtained.

### Eligibility criteria

#### Inclusion criteria

The following inclusion criteria will apply:

• Chemotherapy-naive patients receiving chemotherapy of any emetogenicity level [40].

• >18 years old

• Life expectancy >3 months

• Baseline Karnofsky score >60

• No concurrent neoplasms or illness that induces nausea independent of chemotherapy

• No self-prescribed therapies or complimentary products used for nausea

### Exclusion criteria

The following exclusion criteria will apply:

• Patients requiring radiotherapy

• Pregnant or lactating

• Concurrent use of other ginger-containing supplements and ingestion of large quantities of ginger

• History of adverse reactions to ginger

• Patients with malignancies of gastrointestinal tract/gastrointestinal diseases or nausea and vomiting due to reasons other than chemotherapy

• Thrombocytopenia or patients undergoing chemotherapy that, according to physician discretion, is likely to cause thrombocytopenia (platelets <50 × 10^9/L)

• Currently prescribed warfarin or on anti-coagulant therapy

### Study treatment

#### Ginger extract

The experimental treatment will be a commercial ginger extract manufactured by Bluebonnet Nutrition [41]. This preparation is in capsule form, and is standardised to contain 5% gingerols. Each capsule contains 300 mg of ginger extract with 15 mg of active ingredient per capsule (60 mg per 1.2 g) within white gelatine capsules.

A regimen of 4 capsules per day will be selected in order to incorporate the pharmacokinetics of ginger [36,37].

### Placebo

The placebo capsules will be identical to the ginger capsules in appearance and will contain 300 mg of an inert filler.

### Independent analysis

The ginger capsules will be independently analysed for the active compounds (gingerols and shogaols) by the Southern Cross Plant Science Department at Southern Cross University using a standardised HPLC analysis method by the US Pharmacopeia (USP). Three random samples will be analysed at the beginning of the trial as well as at the end of the trial in order to assess the stability of the bioactive ingredients.

### Concomitant treatment

All anti-emetic medication prescribed by the patient’s medical team, including 5-HT3 antagonists (e.g. ondansetron), corticosteroids (e.g. dexamethasone), and NK1 receptor antagonists (e.g. aprepitant), will be permitted during this trial.

Participants will be advised to avoid consuming large amounts of dietary ginger or additional ginger capsules as well as any other adjuvant or alternative therapy for nausea and vomiting (excluding prescribed anti-emetic medication) during the study period.

Large amounts of ginger is defined as consumption of one serve of either ginger ale, crystallized ginger, or ginger containing meals/products most days (4/7) of the week for the past month; particularly within the week before and during chemotherapy.

### Withdrawal criteria

Any participant who has been randomised and then withdraws will be included in the study on an intention to treat basis with patient consent. If a participant withdraws consent, data will be collected up until their time of withdrawal. Primary outcome data will be collected in these participants where possible.

Any participant who withdraws before being randomised (i.e. allocated to a particular study treatment) will be replaced, so that the next consenting participant receives the randomisation sequence and that participant’s allocated study treatment.

### Study duration

Participants will be enrolled in the study from the time of entry into the trial, through to 4 days after their third chemotherapy session. It is anticipated that it will take one year to recruit the necessary number of participants.

### Treatment assignment and randomisation

Participant numbers will be assigned sequentially to participants as soon as they sign the informed consent form. Participants will be randomly assigned using a computer generated randomisation sequence. The randomisation sequence will be kept separately from the study investigators and will be generated by an independent researcher.

## Methods

### Recruitment

Participants will primarily be recruited through the daily chemotherapy education sessions that are offered by the hospital to patients who have been recently prescribed chemotherapy. Additionally, oncology nursing staff and chemotherapy-scheduling staff will be made aware of the study and will be encouraged to refer patients who may be interested in the study for further screening.

### Screening

Patients will be assessed to ensure that they meet the inclusion and exclusion criteria. All patients who meet the criteria will be invited to participate in the study and be given a participant information sheet. This process may occur at any stage up to seven days prior to chemotherapy.

At the screening, patients will be informed that if they consume large amounts of dietary ginger or additional ginger capsules, as well as any other adjuvant or alternative therapy for nausea and vomiting (excluding prescribed anti-emetic medication), that this should be stopped at least 1 week prior to chemotherapy.

### Questionnaires used

#### Rhodes Inventory of Nausea, Vomiting and Retching (INVR)

The INVR is a self-report questionnaire that measures nausea, vomiting and retching as separate entities based on 8 items with 5-point Likert scales [30]. The frequency and distress of all entities is measured as well as the duration of nausea and the amount of vomitus. The tool is suitable for use during each phase of CINV (i.e anticipatory, acute, and delayed) and is designed to measure symptoms over a 12 hour period; however, for the purpose of this trial, this period was extended to 24 hours to reduce the study burden on patients. It takes less than 5 minutes to complete.

### The Functional Living Index – Emesis – 5 Day Recall (FLIE-5DR)

The FLIE-5DR is a validated nausea and vomiting-specific self-reported outcome measure that investigates the specific impact of chemotherapy-related nausea and vomiting on patients’ activities of daily living [42]. It has 9 items in each of the nausea and vomiting scales, the first item of which rates the extent of nausea or vomiting experienced in the previous 5 days. The remaining items examine patients’ social, recreational and leisure activities, ability to do normal tasks, their enjoyment of eating and drinking, and the hardship caused by their nausea and vomiting on themselves and their carers. Each response is ranked on a seven point scale. The FLIE score is determined by summing the responses to the 9 questions in each scale. Therefore, the range of total scores possible per scale is 9 to 63, with a higher score responding to less hardship and less impact of nausea or vomiting on daily life [42]. No or minimal impact on daily life is defined as an average FLIE item score of no more than 6 on the 7 point scale or a total FLIE score of more than 54 [42]. The FLIE has excellent internal reliability, with a Cronbach’s α > 0.90 for both sub-scales on all assessment points [43,44]. The FLIE takes less than 2 minutes to complete.

### CINV susceptibility questionnaire

This questionnaire has been developed for use in this trial to determine participants’ predisposition to CINV. Previous research has reported several factors correlated with susceptibility to CINV. These include lifestyle factors (e.g. alcohol intake); previous experience of nausea and/or vomiting from causes other than chemotherapy (e.g. motion sickness, pregnancy); and participant characteristics (e.g. age, gender). It is estimated that the questionnaire takes approximately 5 minutes to complete.

### Edmonton Symptom Assessment System (ESAS)

The ESAS is a validated and reliable tool used to assess the severity of common symptoms experienced by cancer patients including pain, anxiety and drowsiness. It includes 10 items that are self-assessed by the patient using individual 10-point scales. This tool has been validated in this population and has reported a Cronbach’s α of 0.79 [45]. The tool will be administrated at -1 day and at 4 days post-chemotherapy for each cycle in order to determine treatment related side-effects. The tool should take approximately 5 minutes to complete.

### Patient Generated - Subjective Global Assessment (PG-SGA)

Nutritional status will be measured using the valid and reliable scored PG-SGA [46]. Using the data gained from this tool, statistical analysis will be conducted to determine the impact of CINV on the participants’ nutritional status. The PG-SGA will be conducted by a dietitian who has undergone training and testing for inter-rater reliability on nutritional status measures. The PG-SGA is specifically designed to assess the nutritional status of cancer patients. This tool provides a global rating of either A (well nourished), B (suspected or moderately malnourished) or C (severely malnourished). This global rating is based upon weight change, dietary intake, GI symptoms, a physical examination and the patient’s functional capacity. A total PG-SGA score is also calculated. A higher score reflects a higher risk of malnutrition and an increased need for nutrition intervention and symptom management.

### Functional Assessment of Cancer Therapy- General (FACT-G) and Fatigue (FACIT-F) subscale

The participants’ self-assessed QoL will be measured using the FACT-G questionnaire, a validated tool that has been widely used in this setting [47]. It contains 27 questions with a 5-point scale, which assesses four domains of patient QoL: physical well-being, social/family well-being, emotional wellbeing, and functional well-being. Strong concurrent validity with the Functional Living Index-Cancer tool was demonstrated with a Pearson coefficient of 0.79 [47]. Additionally, we have included the Functional Assessment of Chronic Illness Therapy- Fatigue (FACIT-F) subscale, a 13 item Likert scale, to assess self-reported symptoms of fatigue before and after each chemotherapy cycle. It is estimated to take between 5-10 minutes to complete.

### Adherence questionnaire

To assess the level of adherence to the study protocol, a questionnaire was developed for patients to record if and when they consumed the ginger/placebo doses per day. This is achieved by either recording the time or marking an X, depending on whether they consumed each dose, in the box corresponding to the dose in question. This is to be completed each day and is expected to take less than 2 minutes to complete.

### Timeline

**Figure 1:**
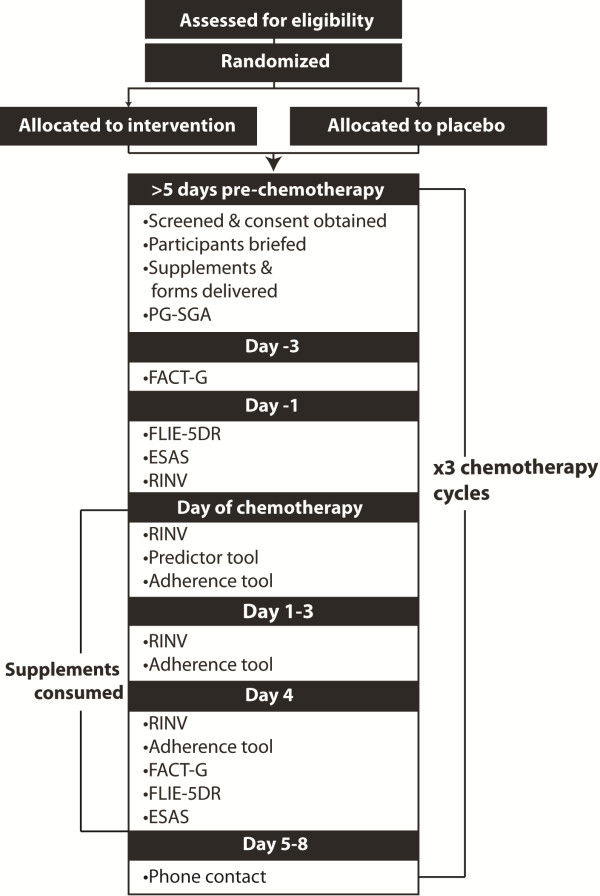
Study flow diagram.

The details of the study procedure are detailed below in chronological order. This timeline contains the details of the study process per cycle and will be repeated for 3 cycles (Figure 1).

### Pre chemotherapy – 7 days prior to chemotherapy

The researcher will see patients as close as possible to 7 days prior to chemotherapy to determine their eligibility. If the patient is a viable candidate, informed consent will be obtained, the details of the study will be explained, and the supplements and questionnaire booklet will be delivered. Participants will be provided with written information and educated regarding the consumption of the supplement.

Participants will be randomised and provided with a 5 day (4 × 300 mg capsules per day) supply of ginger extract (20 capsules) or the placebo control (20 capsules) to be consumed daily with liquid in addition to their usual diet for 4 days, starting on the day of chemotherapy. The supplement will be provided in a sealed plastic container that will be packed by a researcher not involved in data collection.

Participants will be given a booklet containing the self-report questionnaires for CINV, QoL, adverse events, and blinding for the full study period per chemotherapy cycle (5 days). Each booklet will contain:

• One INVR questionnaire per day: one on the day of chemotherapy, and one each of the 4 days post-chemotherapy.

• Two FACT-G/FACIT-F questionnaires per cycle.

• One FLIE-5DR questionnaire per cycle.

• Two ESAS questionnaire.

• One CINV susceptibility questionnaire.

• One Adherence Questionnaire for each day the participant receives the study medication: one on the day of chemotherapy, and one for each of the 4 days post-chemotherapy.

• Instructions on how and when to complete these questionnaires will be included, as well the contact details of the study investigators.

During this consultation, the participant’s nutritional status will also be assessed using the PG-SGA assessment tool.

### Pre chemotherapy – 1 day prior to chemotherapy

The following tools will be completed by the participant 24 hours before chemotherapy:

• One FACT-G/FACIT-F questionnaire

• One ESAS questionnaire

### Day of chemotherapy

The following tools will be completed by the participant on the day of chemotherapy:

• One INVR questionnaire will be completed before chemotherapy commences.

• One CINV susceptibility questionnaire will be completed any time after chemotherapy has commenced.

• One adherence questionnaire.

Additionally, the participant is to consume one ginger/placebo capsule 1 hour before the administration of chemotherapy and then once every 4 hours after that for the remaining 3 capsules. The timing will be discussed with the participant to help ensure the participant understands the regimen.

### Post-chemotherapy – day 1-4 post-chemotherapy

During the 4 days post-chemotherapy, participants will be asked to complete:

• One INVR questionnaire per day. The timing of completion should be at the same time of day as when they completed their previous questionnaire. This will ensure that 24 hours is assessed per questionnaire.

• One adherence questionnaire.

• The participant will consume 4 capsules per day. One before breakfast, one before lunch, one during an afternoon snack, and one before dinner. These capsules are to be consumed one hour before each meal.

### Post-chemotherapy – day 4 post-chemotherapy only

At the end of day 4, participants will be asked to complete:

• One FACT-G/FACIT-F questionnaire

• One FLIE-5DR questionnaire

• One ESAS questionnaire

• One adherence questionnaire

Supplements are consumed using the same schedule as above.

At the end of day 4, participants will no longer be required to consume the oral supplements and any unconsumed supplements, along with the questionnaire booklet, will either be collected by the research team, along with the participant questionnaire booklet, during the participant’s next visit to the hospital or sent directly to the researchers using a reply-paid envelope. Unconsumed supplements of each individual will be counted in order to determine their level of adherence to the study protocol.

### Assessment of blinding

At the end of day 4, the investigator will contact each participant to obtain information regarding the study blinding. This will be determined by asking each participant the following questions: “Do you think you received the placebo or the ginger supplement and why do you think this?”. Participants will also be asked if they have any comments or queries regarding the trial so as to gather feedback for the improvement of the study protocol for future trials.

The timing of the participant’s next chemotherapy cycle will also be discussed and arrangements will be made to meet within the week before chemotherapy in order to dispense additional supplements and assessments.

### Statistical analysis

Analyses will be conducted according to intention-to-treat principles i.e. the consent process will maximise outcome data collection and attempt to assess nausea symptoms for everyone, and will retain original group allocation despite actual compliance levels.

Participants will be block stratified by chemotherapy category (i.e. minimal, low, moderate and high emetogenicity) then randomised within strata into intervention and control groups (Figure 1) [48].

Descriptive statistics will be presented as mean ± standard deviation, or median with range, as appropriate. Parametric analyses will be used for all continuous variables. Chi-square analyses will determine associations between categorical variables. For example, the incidence, severity and type of nausea and vomiting between the two groups. Pearson correlation analysis of continuous variables will be performed. Repeated measures analyses will be conducted to detect between group differences over time as per our statistician recommendations. Statistical significance will be set at p < 0.05 level (two-tailed). Data will be analysed using SPSS for Windows version 22 (Statistical Package for Social Sciences, IL, USA).

### Sample size

A sample size calculation for comparing two means with unpaired t-tests based on the reductions in the prevalence of chemotherapy-induced nausea reported by Panahi et al. [49], estimates that 73 participants would be required in the intervention and control groups (i.e. total of N = 146) to detect this difference with 80 per cent power at the 95 per cent significance level (two tailed).

Approximately 250 patients receive moderately emetogenic chemotherapy and 240 patients receive highly emetogenic chemotherapy at Princess Alexandra Hospital each six months (1/3-1/9/2012) which indicates that the required sample size is obtainable in this study.

### Ethical considerations

The study protocol has been approved by the Metro South Human Research Ethics Committee on the 4th of July, 2013. The trial has also been registered with the Australian New Zealand Clinical Trials Registry and has been assigned the identifier, ACTRN12613000120774. The study complies with the Declaration of Helsinki rules and the principles of Good Clinical Practice guidelines. Informed consent will be gained from all participants before commencing the trial and patient data will be stored securely. Participants will also be monitored for adverse effects and will be discontinued immediately if the study protocol is determined to be causing harm or if the participant chooses to withdraw. This study received grant funding from the Queensland Health – Health Practitioner Scheme.

## Discussion

This study protocol expands on the current literature regarding the efficacy of ginger as an adjuvant therapy for CINV. Recommendations for the use of ginger in the oncology setting are premature, as previous reviews have shown inconsistent results and have possessed several limitations. Primary concerns identified in the literature include the lack of control of anticipatory nausea, the inconsistent use of standardised ginger extracts and validated assessments tools, and a lack of assessment for prognostic factors that may influence individual CINV response [21]. Additionally, recent pharmacokinetic studies demonstrate that the half-life of the active compounds within ginger are relatively short-lived, which suggests that the dosing regimens employed by previous studies may be suboptimal. Furthermore, multiple studies included in these reviews have used anti-emetic therapies that are not congruent with current best practice and anti-emetic guidelines and therefore, the application of these previous findings to current practise are further diminished [21,48].

In designing our trial, we aimed to address these limitations while incorporating elements of rigorous study methodology that have been incorporated in previous trials in this area. For example, our trial will be using multiple, validated assessment tools along with a standardised ginger extract, both of which have been utilised in at least two previous trials [50,51]. We will, however, expand on this by independently analysing our extracts at both the beginning and end of our recruitment phase to ensure consistent potency.

It should also be noted that one study by Ryan et al. [51] found ginger to reduce CINV when ginger supplementation was commenced three days before chemotherapy. We, however, decided against using this methodology and opted for ginger supplementation commencing on the day of chemotherapy due to the following reasons. Firstly, there have been multiple previous trials using ginger for CINV as well as other forms of nausea that did not use the regimen used by Ryan et al. [51] but rather a timeframe and dosage more closely resembling the regimen in our protocol that yielded beneficial results [49,52-54]. In addition to this, to date, there has been no research that has investigated the regimen used in the Ryan et al. [51] study compared to the more typical dosing regimen that has been employed in our study, which restricts one from determining the superiority of said regimen. Lastly, the basis for said regimen, from our research and from the discussion in the Ryan et al. [51] paper, seems to have been implemented largely on a theoretical basis and therefore, until more evidence arises, we have decided to instead opt for the more patient-convenient regimen described in this manuscript.

Our trial will also be the first to introduce multiple novel study design elements. Primarily, our study will be the first to recruit only chemotherapy-naive patients. This strategy should mitigate the significant limitation of anticipatory nausea. It is a response to previous research reporting that CINV control progressively deteriorates with each subsequent chemotherapy cycle, if not adequately controlled during the initial cycle [35]. Due to the association between fatigue and nausea in this setting, we will also investigate the effect that ginger has on this association in order to determine if ginger may be of benefit to patients also experiencing cancer-related fatigue. Finally, our study will also implement a dosing regimen that is consistent with the findings of the previously mentioned pharmacokinetic studies that will likely improve the CINV protection of this therapy. If successful, this trial will provide support for the efficacy of ginger as a viable adjuvant anti-emetic therapy and in doing so, help manage chemotherapy symptoms and assist in improving patient QoL.

## Abbreviations

CINV: Chemotherapy-induced Nausea and vomiting; QoL: Quality of life; PG-SGA: Patient generated - subjective global assessment; FACT-G: Functional assessment of cancer therapy- general; FACIT-F: Functional assessment of chronic illness therapy-fatigue; INVR: Rhodes inventory of Nausea vomiting and retching; ESAS: Edmonton Symptom Assessment System; FLIE-5DR: The functional living Index – Emesis – 5 day recall.

## Competing interests

Luis Vitetta has received National Institute of Complementary Medicine and National Health and Medical Research Council of Australia competitive funding and Industry support for research into nutraceuticals. No other author has any competing interests to disclose.

## Authors’ contributions

WM was responsible for the development of the manuscript, study design, and ethics submission, EI is the principle investigator, provided PhD supervision, management of funding, hiring of research assistant, and overall supervision of project progression, LV participated in manuscript development, product acquisition, provided PhD supervision, and information regarding the mechanism of action of ginger. KR provided PhD supervision and participated in the development of the study design and manuscript development, ALM participated in the development of the study design, provided information regarding prognostic factors, and contributed to the development of the protocol manuscript. DT contributed with study design and development of manuscript as well providing background on emetogenic chemotherapy regimens. AS provided product knowledge, PhD supervision and participated in the design of the study. DM provided information regarding the safety of ginger supplementation, contributed to the development of the protocol manuscript with the manuscript development as well as the procurement of placebo capsules. All authors read and approved the final manuscript.

## Pre-publication history

The pre-publication history for this paper can be accessed here:

http://www.biomedcentral.com/1472-6882/14/134/prepub
